# *Proceedings B* 2021: the year in review

**DOI:** 10.1098/rspb.2021.2586

**Published:** 2022-01-12

**Authors:** Spencer C. H. Barrett

**Affiliations:** *Editor-in-Chief*

As I write my annual review of the year at *Proceedings B*, the breaking news of the new COVID variant of concern B.1.1.529 ‘Omicron’ is dominating the headlines and leading us to think ‘here we go again’. Despite considerable success in vaccination campaigns in some countries, the COVID pandemic will be a recurring constraint on our lives until vaccine supply and inequality issues are solved and a significant proportion of the global population is vaccinated. So, although I had hoped in my editorial for 2020 that this year might see a significant reduction in the disruptions that we experienced last year, the latest news suggests that masks, social distancing, travel restrictions, and video-conferencing are likely to be with us for some time. Despite these ongoing challenges, *Proceedings B* continues to weather the storm and we have adjusted to pandemic restrictions effectively. Although journal staff at the Royal Society were allowed into 6–9 Carlton House Terrace towards the end of 2021, most still prefer to work from home and thus avoid commuting into London. This has in no way influenced the editorial process and every two weeks throughout 2021 *Proceedings B* has been able to showcase the latest findings in organismal biology. Although our submissions in 2021 dropped by roughly 8% compared with the preceding year, a pattern evident across all Royal Society journals, in 2020 we experienced an equivalent bounce in submissions; perhaps as a consequence of the first year of the pandemic. As a result, we are currently now at pre-COVID submission levels.

From 1 January to 31 October 2021 we received 2276 submissions, a decrease of 339 articles in comparison with the same period in 2020. Of these submissions, the proportion that was rejected was very similar to the preceding year (2020—75%; 2021—74%). So far this year, 505 articles have been accepted for publication and of these 215 are open access, a significant increase over 2020 when we published 132 in this category. This upward trend is encouraging because of the recent decision by the Royal Society Publishing Board to make the transition to full open access for *Proceedings B* within a few years (see below). Our times (in median days) to first decision (15), final decision (60), and from submission receipt to publication (88) have remained steady and are within target times. Our current projections are approximately 2730 submissions by the end of 2021. We received submissions from numerous geographical regions, with the largest numbers to date coming from the USA (587), UK (286), China (234), Australia (141), Germany (126), Canada (115), Japan (90), France (76), Spain (64), and Sweden (52). Like many international science journals, we struggle to attract submissions from certain global regions, such as South America, Africa, and the Indian subcontinent, and we encourage those of you who have collaborators and colleagues in these regions to consider suggesting that they submit their work to *Proceedings B*. The impact factor for *Proceedings B* improved in 2021, rising from 4.637 in the previous year to 5.349, and the journal is ranked 13th out of 93 journals in the Journal Citation Reports category for ‘Biology’.

Review articles written for a general audience are published in every issue of *Proceedings B* and remain a popular forum for providing timely overviews of ‘hot areas’ in the biological sciences. Reviews editor Innes Cuthill has done an excellent job of maintaining a steady flow of reviews on diverse topics, and during 2021 he received 93 proposals of which 73 have been approved for submission and 19 have been published. Our biannual review by the President's Award Winner of the *Canadian Society for Ecology and Evolution* was ‘Network ecology in dynamic landscapes' [1] by Marie-Josée Fortin (University of Toronto) and colleagues Mark Dale (University of Northern British Columbia) and Chris Brimacombe (University of Toronto). The article provides a unifying framework for ecological network dynamics, particularly in relation to environmental change. The framework they propose emphasizes the importance of the interplay between species interaction networks and the spatial distribution of habitat patches for identifying which features of networks are critical for maintaining species interactions when landscapes are continually changing. The approach helps to identify the combination of network properties that influences the balance between positive effects, such as rescue through dispersal and gene flow, and negative effects involving disturbance, species invasions, and predation. The ideas presented by Fortin and colleagues are of considerable value to ecologists and landscape and wildlife managers because they enable predictions to be made about the complex dynamics of species interactions across different spatial scales, trophic levels, and ecosystem functions.

This year's annual Darwin review in *Proceedings B*, ‘The stagnation paradox: the ever-improving but (more or less) stationary population fitness' [2] by Hanna Kokko (University of Zurich) is concerned with the fascinating topic of the evolution of population fitness and highlights why an unambiguous measure of population fitness does not appear to exist. Beginning with Fisher's fundamental theorem [3], Kokko shows how fitness can improve over time but still stay constant, a situation she refers to as the ‘stagnation paradox’. By taking us through a series of definitional minefields, as well as specific empirical (e.g. long-term evolution studies of bacterial populations) and conceptual (Fisher's concept of environmental deterioration, life-history theory, sexual conflict) examples, she shows how the paradox can be resolved. Her article concludes with a strong plea for the integration of demographic studies and eco-evolutionary feedbacks into microevolutionary, and perhaps also macroevolutionary studies.

This year we commissioned two Special Features in *Proceedings B*, which involve collections of articles on a single theme chosen by the Editors based on the submission of proposals. Both will appear shortly. ‘Evolution in changing seas' edited by Katie E. Lotteros, Molly Albecker, and Geoffrey Trussell (Northeastern University Marine Science Center) advances our understanding of evolutionary change in marine systems. A particular focus of the special feature is on determining the appropriate experimental designs and statistical approaches for robustly testing hypotheses in an environment that often presents challenging logistical problems for data collection. Papers encompass examples across the tree of life and help to bridge knowledge gaps on the evolutionary biology of marine systems. Our second special feature ‘Stability and manoeuvrability in animal movement: lessons from biology, modelling and robotics edited by Andrew Biewener (Harvard University), Richard Bomphrey (Royal Veterinary College), Monica Daley (University of California, Irvine), and Auke Ijspeert (Swiss Federal Institute of Technology, Lausanne) concerns the integration of engineering approaches for solving the problem of how animals achieve stability in their movement through complex environments. Through modelling and experiments, including the use of diverse bioinspired robots, new insights on behaviours associated with predator–prey interactions, mating, obstacle avoidance, and navigation are being discovered using these approaches.

We continue to broaden the scope of articles published in *Proceedings B* and during 2021 we introduced a new subject area for research articles—*Biological Applications*—concerned with the application of concepts in the biological sciences for solving problems of environmental and societal relevance. We introduced this category to take advantage of the burgeoning growth in applied research of relevance to policymakers and managers of biological resources. We encourage submissions on a wide spectrum of topics including agroecosystems, biotechnology, genetic resources, food security, forestry, fisheries and aquaculture, wildlife biology, restoration ecology, land use management, invasion biology, human health and epidemiology, environmental pollution, and pest, disease, and weed control. For submissions to be successful they should integrate basic biological concepts and their practical applications in a question- or hypothesis-driven framework. Authors must also indicate explicitly how their results have practical implications for the problems they are addressing and also their general relevance. Submissions that are too specialized focusing narrowly on a particular system or case study are not encouraged. We particularly welcome interdisciplinary studies, and in common with other research articles in *Proceedings B*, submissions should be of outstanding scientific importance and broad general interest and follow our standard requirements for research articles. This new subject area has proven to be popular with our authors and to date we have received 111 articles making up 5% of all submissions to *Proceedings B*.

Two additional categories of articles that we recently introduced—*Evidence Synthesis* and *Biological Science Practices*—continue to attract interesting papers, several of which have received considerable media attention. So far there have been 71 submissions, 17 of which have been published in the *Evidence Synthesis* article type, and 11 in *Biological Science Practices*. Online collections of published articles in these two categories are available at: https://royalsocietypublishing.org/topic/special-collections/evidence-synthesis and https://royalsocietypublishing.org/rspb/biological-science-practices. These collections provide ample testimony to the increasing diversification of content in *Proceedings B* and illustrate how the journal is transitioning from its former status as primarily an ecology, evolution, and behaviour journal to one that now includes articles on all aspects of fundamental and applied biology as well as how biological science is practised and its policy and management implications.

Maurine Neiman (University of Iowa) continues with her work as Preprint Editor for the journal taking advantage of the submission of unpublished articles to the preprint server *bioRxiv*. This initiative, the first among Royal Society journals, has proven to be highly successful and Maurine has expanded her Preprint Editorial Team to include around 30 members from many different countries with a particular focus on early career scientists. In 2021, 45 papers have been submitted to the journal via the preprint route, 17 have been accepted, 18 rejected, and 10 are still in review. For those interested in reading an article on the preprint solicitation process, how it originated and how it is implemented at *Proceedings B*, a valuable summary was written by Maurine and her colleagues which was recently published in our *Biological Science Practices* article category [4] https://royalsocietypublishing.org/doi/10.1098/rspb.2021.1248.

The Royal Society Publishing Board has recently agreed that the Society's primary research journals should aim to make the transition to full open access mode over the next 5 years, following a recommendation by the Council of the Royal Society. This means that *Proceedings B* along with *Proceedings A*, *Biology Letters*, and *Interface* should have no subscriptions or access controls for their 2026 volumes. It is proposed that progress toward this objective will be made in two main ways: (i) a gradual increase in the number of authors individually opting for open access publication, as seen since 2006 and especially this year, as awareness and funder support for open access and article processing charges (APCs) grow; (ii) the continued development of transitional ‘read and publish’ type arrangements with institutions and consortia around the world. This second mechanism is a new one but the Publishing Board has negotiated several agreements for 2022 and there is a strong pipeline of further deals at various stages of development. These transitional deals will have the effect of a more rapid, stepwise increase in open access take-up as the authors in the eligible institutions are able to take advantage of unlimited, APC-free open access to the journals. A waiver policy will also be in place for authors from disadvantaged countries and for those without funding.

To wrap up this year's editorial I extend my sincerest thanks to all members of the Editorial Board for their hard work in making *Proceedings B* a ‘must-read’ for biologists interested in breaking discoveries in the biological sciences. A particular thanks is in order to those Associate Editors who will retire at the end of 2021. I hope that your work with *Proceedings B* has been challenging but rewarding and has provided opportunities to communicate and develop friendships with other board members and staff. A striking feature of the Editorial Board of *Proceedings B* is that the vast majority of those whom we initially contact accept our offer, with many choosing to stay on for a second term. This year we successfully recruited 16 new Associate Editors, 62% of whom are female, thus maintaining the near gender parity (48% female) on our Editorial Board.

There is no doubt that part of the satisfaction in working for *Proceedings B* as a board member arises from the professionalism of the office staff in London who make the job a lot less arduous than might be initially envisioned. I would like to thank our Editorial Team in the London office, consisting of Editorial Coordinators Jennifer Kren and Callum Shoosmith, and Production Editor Simon Clackson, for their conscientious work in making sure that *Proceedings B* runs efficiently. Lastly, I would again like to thank the Publishing Editor of *Proceedings B*, Shalene Singh-Shepherd, to whom I am especially grateful. Her dedication and wise counsel to me and all the Editorial Board has been exemplary, especially given that we have all experienced another challenging year.
